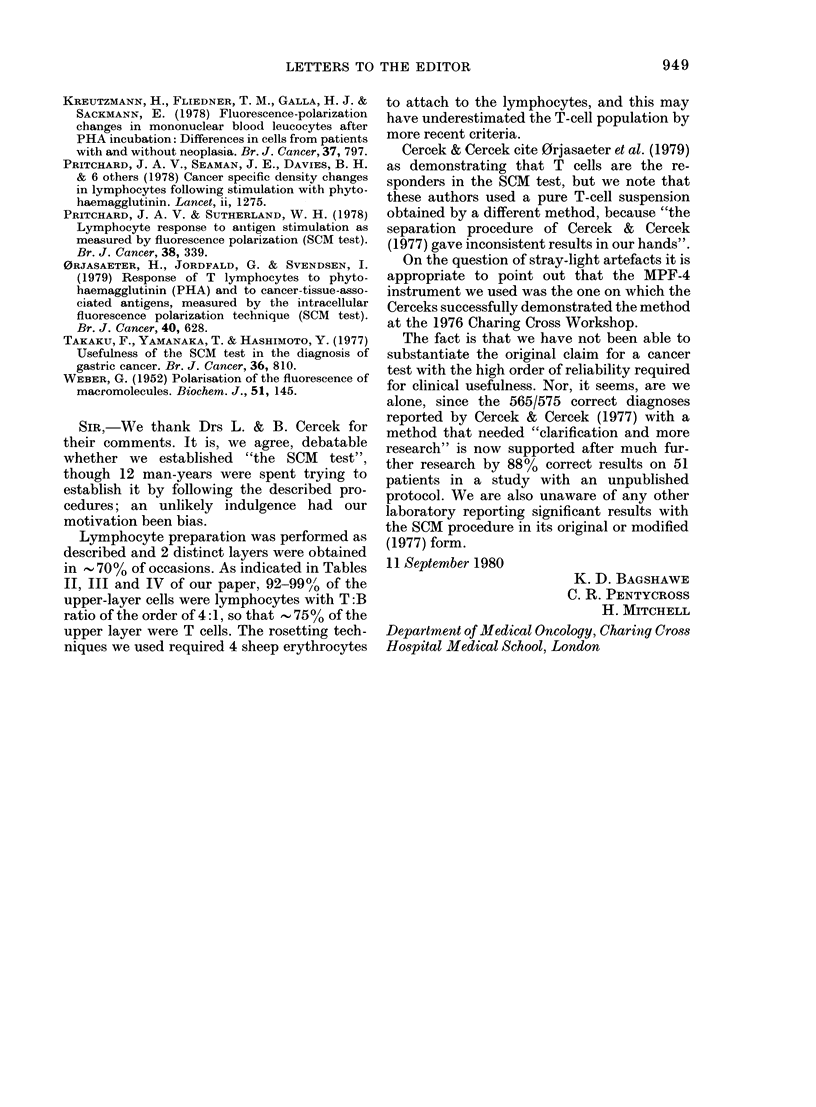# Comments on “The SCM Test for Cancer. An Evaluation in Terms of Lymphocytes from Healthy Donors and Cancer Patients”

**Published:** 1980-12

**Authors:** K. D. Bagshawe, C. R. Pentycross, H. Mitchell


Sir,—We thank Drs L. & B. Cercek for their comments. It is, we agree, debatable whether we established "the SCM test", though 12 man-years were spent trying to establish it by following the described procedures; an unlikely indulgence had our motivation been bias.

Lymphocyte preparation was performed as described and 2 distinct layers were obtained in ~70% of occasions. As indicated in Tables II, III and IV of our paper, 92–99% of the upper-layer cells were lymphocytes with T:B ratio of the order of 4:1, so that ~75% of the upper layer were T cells. The rosetting techniques we used required 4 sheep erythrocytes to attach to the lymphocytes, and this may have underestimated the T-cell population by more recent criteria.

Cercek & Cercek cite Ørjasaeter *et al.* (1979) as demonstrating that T cells are the responders in the SCM test, but we note that these authors used a pure T-cell suspension obtained by a different method, because "the separation procedure of Cercek & Cercek (1977) gave inconsistent results in our hands".

On the question of stray-light artefacts it is appropriate to point out that the MPF-4 instrument we used was the one on which the Cerceks successfully demonstrated the method at the 1976 Charing Cross Workshop.

The fact is that we have not been able to substantiate the original claim for a cancer test with the high order of reliability required for clinical usefulness. Nor, it seems, are we alone, since the 565/575 correct diagnoses reported by Cercek & Cercek (1977) with a method that needed "clarification and more research" is now supported after much further research by 88% correct results on 51 patients in a study with an unpublished protocol. We are also unaware of any other laboratory reporting significant results with the SCM procedure in its original or modified (1977) form.

11 *September* 1980

K. D. Bagshawe
C. R. Pentycross
H. Mitchell

*Department of Medical Oncology, Charing Cross Hospital Medical School, London*